# Boosting for insight and/or boosting for agency? How to maximize accurate test interpretation with natural frequencies

**DOI:** 10.1186/s12909-023-04025-6

**Published:** 2023-02-07

**Authors:** Markus A. Feufel, Niklas Keller, Friederike Kendel, Claudia D. Spies

**Affiliations:** 1 Division of Ergonomics in the Department of Psychology and Ergonomics (IPA), Technische Universität Berlin, Straße des 17. Juni 135, 10623 Berlin, Germany; 2 Present Address: Simply Rational GmbH, Berlin, Germany; 3 Present Address: Institute for Gender in Medicine at Charité - Universitätsmedizin Berlin, Freie Universität Berlin, Humboldt-Universität zu Berlin, and Berlin Institute of Health, Berlin, Germany; 4 Present Address: Department of Anesthesiology and Operative Intensive Care Medicine at Charité - Universitätsmedizin Berlin, Freie Universität Berlin, Humboldt-Universität zu Berlin, and Berlin Institute of Health, Berlin, Germany

**Keywords:** Test interpretation, Bayesian reasoning, Natural frequencies, Boosting, Nudging

## Abstract

**Background:**

Many physicians do not know how to accurately interpret test results using Bayes’ rule. As a remedy, two kinds of interventions have been shown effective: boosting insight and boosting agency with natural frequencies. To boost insight, test statistics are provided in natural frequencies (rather than conditional probabilities), without instructions on how to use them. To boost agency, a training is provided on how to translate probabilities into natural frequencies and apply them in Bayes’ rule. What has not been shown is whether boosting agency is sufficient or if representing test statistics in natural frequencies may additionally boost insight to maximize accurate test interpretation.

**Methods:**

We used a pre/posttest design to assess test interpretation accuracy of 577 medical students before and after a training on two Bayesian reasoning tasks, one providing conditional probabilities, the other natural frequencies. The pretest assessed baseline abilities versus the effect of natural frequencies to boost insight. After participants received a training on how to translate conditional probabilities into natural frequencies and how to apply them in Bayes’ rule, test interpretation skills were assessed using the same tasks again, comparing the effects of training-induced agency with versus without additionally boosting insight (i.e., test statistics in natural frequencies versus conditional probabilities).

**Results:**

Compared to the test question formatted in conditional probabilities (34% correct answers), natural frequencies facilitated Bayesian reasoning without training (68%), that is, they increased insight. The training on how to use natural frequencies improved performance for tasks formatted in conditional probabilities (64%). Performance was maximal after training and with test statistics formatted in natural frequencies, that is, with a combination of boosting insight *and* agency (89%).

**Conclusions:**

Natural frequencies should be used to boost insight *and* agency to maximize effective use of teaching resources. Thus, mandating that test statistics are provided in natural frequencies *and* adopting short trainings on how to translate conditional probabilities into natural frequencies and how to apply them in Bayes’ rule will help to maximize accurate test interpretation.

**Trial registration:**

The study was a registered with the German Clinical Trial Registry (DRKS00008723; 06/03/2015).

To increase “individuals’ own decision-making competences” [1], two educational approaches have been differentiated in the behavioral sciences: boosting of insight and boosting of agency. Whereas the former approach refers to information formats that facilitate retrieval of decision-relevant information and increase people’s *insight* in how to apply them without instruction, the latter refers to trainings that convey specific problem-solving skills to strengthen people’s *agency* to implement a certain behavior or decision.^1^ In this article, we apply this distinction to interventions aimed at improving accurate medical test interpretation. Specifically, we assume that the statistical format of *natural frequencies* [2] can be used to boost insight (to represent test statistics in an easy-to-understand format that facilitates test interpretation without instructions), to boost agency (to explain how to use test statistics to interpret test results), or both (when used to represent test statistics *and* explain how to use them). Rather than siding with any single approach, we suggest that all three approaches have value. The question is when to use which to maximize effective use of teaching resources [3].

To improve Bayesian reasoning, which is needed for accurate test interpretation, natural frequencies have been successfully implemented to boost both insight and agency. Since Gigerenzer and Hoffrage published their seminal paper in 1995, a meta-analysis of 35 articles has shown that representing test statistics as natural frequencies rather than conditional probabilities can “improve Bayesian reasoning without instructions,” that is natural frequencies boost insight [2, 4–6]. Follow-up studies demonstrated that natural frequencies can also be used to boost agency and “teach Bayesian reasoning in less than two hours” [7–14] and that, as a consequence, “the degree of improvement was. .. larger than. .. without training, that is, by merely presenting information in natural frequencies” [7]. What has not been shown, however, is whether using natural frequencies as a combination to boost both insight *and* agency is more effective than either intervention by itself. In addition to replicating the effects of natural frequencies with and without instruction, the primary goal of this paper is thus to determine whether natural frequencies should be used to boost insight, agency, or both to maximize the effectiveness of teaching test interpretation skills to future health professionals. We shortly explain the rationales underlying accurate test interpretation before we present the study and discuss implications for medical education.

## Background

A woman with a positive mammogram will want to know: Does that mean I have cancer? Studies have repeatedly shown that a majority of physicians do not know how to calculate the probability of a disease given a positive test result, that is, the test’s *positive predictive value* or PPV [15–17]. If physicians lack these basic statistical skills, evidence-based medicine (EBM) and accurate risk communication with patients remain illusory.

To calculate PPVs, physicians should adhere to *Bayes’ rule*, a formula that prescribes how to correctly combine the a priori prevalence of the disease to be tested with the likelihood of the test to yield correct positive results (sensitivity) and false positive results (1–specificity). If we assume that the sensitivity of a mammogram is 90%, the false positive rate is 9%, and the prevalence of breast cancer is 1% (i.e., 99% of the women have no breast cancer), Bayes’ rule prescribes the following calculation:

$$PPV=\frac{prevalence\ x\ sensitivity}{prevalence\ x\ sensitivity+\left(\left(1- prevelance\right)\ x\ false- positive\ rate\right)}$$

$$PPV=\frac{.01\ x.9}{.01\ x.9+.99\ x.09}\approx 9\%$$

In 1995, Gigerenzer and Hoffrage showed that representing conditional probabilities (e.g., sensitivity, specificity) as natural frequencies, defined as frequency counts that preserve base rates, facilitates Bayesian reasoning. Applied to the mammogram example, we obtain:

- 1% prevalence translates as “Of 1,000 women, 10 have breast cancer, 990 do not”.
- 90% sensitivity as “Of 10 women with cancer, 9 will test correct positive”.
- 9% false-positive-rate as “Of 990 healthy women, 89 will test false positive”.

**Fig. 1:**
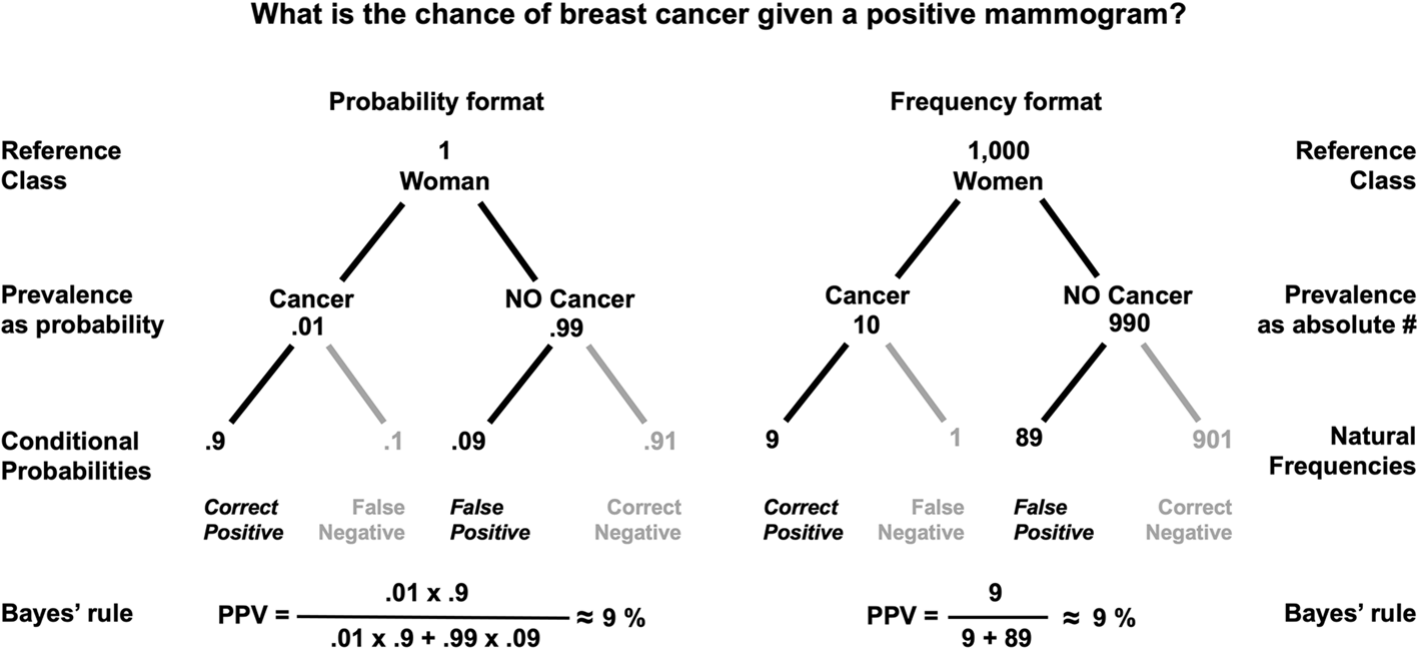
Comparison of two statistical formats for calculating the positive predictive value (PPV) of a mammogram for women in the general population. The left tree shows four *conditional probabilities* at the lowest level compared to four *natural frequencies* in the tree on the right. Whereas the conditional probabilities are normalized, that is, they do not add up to 100%, the natural frequencies are not and add up to the reference class of 9 + 1 + 89 + 901 = 1,000 women. This difference simplifies Bayesian calculations at the bottom of the Figure needed to infer the PPV of the test. Note. Numbers are rounded.

Importantly, when inserted into Bayes’ rule, the results are identical with both formats as shown in Fig. 1. However, computations are simplified and require fewer steps with natural frequencies because they preserve base rates (i.e., the natural frequencies at the lowest level of the tree in Fig. 1 add up to the reference class of 9 + 1 + 89 + 901 = 1,000 women) and can be directly entered into Bayes’ formula without requiring additional calculations.

As stated above, recent meta-analyses have shown that test statistics that are presented in natural frequencies rather than conditional probabilities reliably boost insight and “improve Bayesian reasoning without instructions” [4–6]. Moreover, several training studies using natural frequencies to boost agency [7–14] showed that “the degree of improvement was. .. larger than. .. without training” [7]. What is not known, however, is whether a training on how to use natural frequencies is sufficient to boost medical students’ Bayesian reasoning skills or if representing test statistics in natural frequencies rather than conditional probabilities can additionally boost their insight into the test statistics and how to use them. We test the following hypotheses:

H1: When natural frequencies are used to boost insight into relevant test statistics, test interpretation will be more accurate than with conditional probabilities (i.e., without training). H1 replicates existing evidence concerning the insight-inducing effect of natural frequencies without training [5, 6].

H2: When natural frequencies are used to boost agency, test interpretation with conditional probabilities (the training only condition) will be a) more accurate than conditional probabilities without training (because students will have learned how to translate probabilities into frequencies) (H2a) and b) more accurate than natural frequencies without training (because they will also have learned how to directly enter the latter to facilitate Bayesian calculations) (H2b). That is, H2a replicates the established effect of training (a boost of agency without an additional boost of insight), whereas H2b suggests that training (a boost of agency) is superior to providing natural frequencies (a boost of insight) alone [7, 8].

H3: When natural frequencies are used to boost both insight *and* agency, test interpretation will be maximally accurate, because natural frequencies do not have to first be calculated (insight) and students will know how to directly enter them into Bayes’ rule (agency).

## Methods

To develop the boost of agency for our study, we followed previous training studies, most of which used some kind of visual aid to convey the principles underlying natural frequencies or, more generally, how to represent test statistics so that the relationships between the prevalence and the number of correct positives and false positives become evident [7–14]. Although visualizations of natural frequencies seem to improve accurate test interpretation in general [6, 7, 10, 11, 18], there is thus far mixed evidence concerning which visualization works best [19–21]. Thus, we relied on the most commonly used visual representation so far and designed a 1-hour training that “showed participants how to translate probability information into. .. a frequency tree” [7]. As part of the training intervention, medical students were also taught how to use these natural frequency trees to implement Bayes’ rule and calculate PPVs und negative predictive values (NPV) for common medical tests. To enhance the external validity and students’ motivation to participate in the training, we chose tests that reflected ongoing course work and provided actual estimates for test sensitivities and specificities and the population-specific prevalence of the diseases to be tested. The training format is designed for groups of up to 20 participants and consists of the following four steps (for a detailed description see Table 1):

1. a 5-minute introduction to Bayes’ rule and how to apply it to medical test interpretation, the challenges to calculate test statistics using conditional probabilities, and the advantages of using natural frequencies.
2. a 5-minute explanation of how to translate conditional probabilities into natural frequencies using frequency trees and how to use them to implement Bayes’ rule and calculate PPVs and NPVs of medical tests.
3. a 30-minute practical exercise where students apply the principles and frequency tree representations learned in Step 1 and 2 to calculate PPVs and NPVs for actual medical tests and patient populations (selected to match students’ course work).
4. a 20-minute interactive presentation of the results from Step 3 and discussion of potential implications for medical practice and the communication of test results with patients.

**Table 1:** Steps, content, resources, and instructions of the training intervention aimed at boosting agency for accurate test interpretation

Steps	Content	Resources used ^**a**^	Training Instructions
Pretest (immediately before training)	Untimed assessment of Bayesian reasoning with natural frequencies and conditional probabilities	the 2 Bayesian problems in the Berlin Numeracy Test [22]	Clarify that the test is anonymous, voluntary, and used to evaluate the training, NOT participants’ performance.
Step 1 Introduction (all participants; 5 minutes)	Lecture on the relevance of Bayesian reasoning for everyday medical practice, the challenges posed by conditional probabilities, and the advantages of natural frequencies	Presentation based on [4, 7, 17]	a Recent examples of tests that attracted media attention may increase participants’ interest b Point out that the problem is not a lack of mathematical skills, but the way information is formatted
Step 2 Explanation (all participants; 5 minutes)	Explanation of how to translate conditional probabilities into natural frequencies and how to implement them into Bayes’ rule using concrete examples	Presentation based on [4, 7, 17]	a Emphasize the relationship between prevalence and PPV / NPV for a given test b Examples highlight that most population screening tests are good at excluding, NOT at diagnosing diseases due to low disease prevalence (low PPV; high NPV).
Step 3 Practical exercise (groups of four; 30 minutes)	Participants calculate PPV / NPV given sensitivity/specificity of common medical tests and disease prevalence based on principles from Steps 1 and 2	• Exercise Sheet providing test statistics and natural frequency trees to fill-in • Pocket calculator	a To enhance motivation, use actual test statistics and the prevalence for populations that match participants’ interests / expertise. b When creating frequency trees, start with sufficiently large numbers to facilitate calculation using integers.
Step 4 Interactive presentation and discussion (all participants; 20 Minutes)	Participants present results of Step 3 and interactively discuss the relevance of test interpretation to everyday medical practice and communication with patients	White Board or Flip Chart	a Taking a patient perspective may help to elicit discussions on ethical implications of medical testing, statistics, and interpretation. b Note that clinical experience is important to gauge the need for medical testing and estimate prevalence in subpopulations.
Posttest (immediately after training)	Untimed re-assessment of Bayesian reasoning with natural frequencies and conditional probabilities	Same as Pretest	Same as Pretest

^a^ All materials are available on request from the authors

Abbreviations. PPV = positive predictive value. NPV = negative predictive value

To test the hypotheses, we used a within-subjects 2 × 2 design with test questions being administered in two formats (conditional probability versus natural frequencies) and either with or without the training intervention. Specifically, we used a pre-posttest to assess medical students’ Bayesian reasoning skills immediately before and after the 1-hour training session, using the same two Bayesian reasoning problems, one in natural frequencies, the other in conditional probabilities (see Table 2). Both problems are taken from the Berlin Numeracy Test, a 4-item test^2^ validated to assess people’s untimed ability to reason with numbers (i.e., their numeracy [22];) and test Bayesian reasoning in a non-medical domain, measuring transfer learning. That is, they measure the ability to apply general principles rather than to replicate domain-specific solutions acquired during the training. Also, because performance is typically worse if different tasks are used for training and testing, the chosen test questions should provide a strong test of the effects of the training intervention [23]. Participants received the questions in the same order during both pre- and posttest and could take as much time as they needed to solve them.

**Table 2:** Bayesian reasoning tasks from the Berlin Numeracy Test [22] used for pre- and posttest

Bayesian reasoning task with natural frequencies	Answer option
Out of 1,000 people in a small town, 500 are members of a choir. Out of these 500 members in a choir, 100 are men. Out of the 500 inhabitants that are not in a choir, 300 are men. What is the probability that a randomly drawn man is a member of the choir? Please indicate the probability in percent.	________%
Bayesian reasoning task with conditional probabilities	Answer option
In a forest 20% of mushrooms are red, 50% brown and 30% white. A red mushroom is poisonous with a probability of 20%. A mushroom that is not red is poisonous with a probability of 5%. What is the probability that a poisonous mushroom in the forest is red?	________%

The pretest was designed to test H1 by replicating the insight-boosting effect of representing test statistics as natural frequencies (the insight condition) versus conditional probabilities (the baseline condition; see [4–6]) without training. The posttest with conditional probabilities was designed to test H2, that is, whether using natural frequencies as a training (the agency condition) helps participants outperform both their pretest with conditional probabilities (i.e., the baseline condition, testing the effect of training alone; H2a) and the pretest with natural frequencies (i.e., comparing the effect of training with the insight-boosting effect of natural frequencies without training; H2b) [7, 8]. Finally, the posttest with natural frequencies was intended to test H3, that is, whether the combination of boosting both insight *and* agency provides an additional benefit over the agency condition (i.e., conditional probabilities after the training) and the insight condition (i.e., providing natural frequencies before the training) alone.

Participants were recruited between 2015 and 2016 from three consecutive cohorts, all of which were in their 5th, and final, year of undergraduate medical training. To our knowledge, the medical curriculum contained no prior systematic training of Bayes rule and how to apply it to test interpretation, but their prior knowledge of statistical reasoning likely differed based on what they had learned about probability theory in high school. The study took place as part of a hands-on seminar about statistical reasoning for medical students. Instructors were recruited from various institutes of the university hospital and had not interacted with the students on this topic prior to the seminar. Whereas participation in the seminar was compulsory as part of the medical curriculum, participation in the study was voluntary. No incentives were provided for participation. The study was approved by the medical school’s data protection authorities and ethics committee (EA/067/15; 12/11/2014) and registered with the German Clinical Trial Registry (DRKS00008723; 06/03/2015).

Out of the 822 students in the three cohorts, 203 (25%) did not attend the classes or did not participate. Of the remaining 619 (75%) students, 42 participated only once, leaving 577 students (70%) who participated during both pretest and posttest to be included in the analysis.^3^ A total of 67 (12%) of the 577 participants did not report age or sex. Of the remaining 510 students, 61% were female with a mean age = 25.2 (SD = 3.1; range = 21–42). 10 participants missed to answer one of the two test questions (one person missed the question with conditional probabilities during both pre and posttest; six did not answer the question with conditional probabilities during the posttest; during the pretest, two participants missed a question with natural frequencies; one other person missed the question with condition probabilities). We performed analyses with and without these participants and counting the void answers as missing or incorrect, but the pattern of the results remained identical. For the data we present here, we provide the most conservative estimate of effectiveness and count the missing answers as incorrect.

To test H1 and H2, we ran planned comparisons of the respective conditions in a binomial generalized linear mixed model with the within-subject manipulations (boosts for insight and/or agency) as fixed effects and participant ID as a random effect with varying intercept. To assess H3, we tested the interaction of the main effects of boosting insight and agency. To do so, we used R (version 4.1.2), tidyverse (version 1.3.1) to prepare the dataset, lme 4 (version 1.1–27.1) to model the data, and multcomp (version 1.4–19) to setup the contrasts. We also report the corresponding odds ratios (OR) as a measure of effect size. To exclude regression to the mean effects as possible explanation of the observed differences in pre-posttest designs [24], we report the proportion of participants showing improved versus worse performance in the posttest. Descriptive analyses were performed and plotted using Microsoft Excel for Mac Version 16.57 (Microsoft Corp, Redmond, Washington).

## Results

The analysis showed that, before the training, the percentage of students providing the correct answers was about 30 points higher for the task formatted in natural frequencies (68%) versus conditional probabilities (36%) (*β* = 2.45, SE = 0.19, *P* < .001), supporting H1. The corresponding Odds Ratio (OR) suggests that the odds that students provided a correct answer when given natural frequencies increased 11-fold compared to the task formatted in conditional probabilities (OR = 11.55, 95% CI [7.92, 16.87]).

After the 1-hour training intervention, the percentage of students with correct answers increased for the test question with conditional probabilities from 36% during the pretest to 64% during the posttest (*β* = 2.14, SE = 0.19, *P* < .001), confirming H2a. The odds of a correct answer increased about 8-fold after the training (OR = 8.53, 95% CI [5.93, 12.28]) and the proportion of improved answers was 173/577 (30%) compared to 8/577 (1.4%) cases with decreased performance after the training. However, students’ performance on the posttest question in conditional probabilities (64%) and the pretest question in natural frequencies (68%) did not differ (*β* = − 0.30, SE = 0.17, *P* = .069; OR = 0.74, 95% CI [0.532, 1.02]). This disconfirms H2b.

**Fig. 2:**
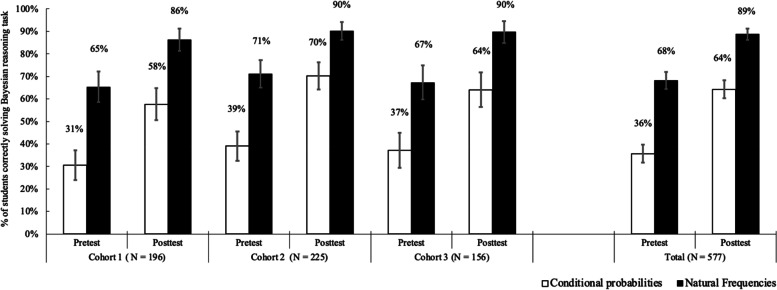
Percent of students correctly solving Bayesian reasoning tasks (*N* = 577) before (pretest) and after a training boost (posttest). Data are presented separately for each task format (conditional probabilities = outlined bars; natural frequencies = solid bars) and each of three student cohorts trained during three consecutive courses. Error bars reflect two standard errors above and below the observed proportions.

Finally, we found main effects of both statistical format (*β* = 1.27, SE = 0.09, *P* < .001; OR = 3.55, 95% CI [2.96, 4.26]) and training (*β* = 1.10, SE = 0.09, *P* < .001; OR = 3.01, 95% CI [2.53,3.58]). The interaction was not significant (*β* = − 0.02, SE = 0.07, *P* = .759; OR = 0.98, 95% CI [0.84, 1.13]), but performance was maximal after the training and for the test question framed in natural frequencies (*β* = 3.07, SE = 0.21, *P* < .001), with the odds of a correct answer increasing about 21-fold compared to all other pretest and posttest conditions (OR = 21.5, 95% CI [14.17, 32.63]). This supports H3. Descriptively, performance on the question using natural frequencies increased from 68% during the pretest to 89% during the posttest, where the proportion of participants with improved performance was 127/577 (22%) compared to 8/577 (1.4%) showing worse performance. The combined condition also outperformed the posttest question with conditional probabilities with 89% to 64% correct answers. Figure 2 shows that the pattern of results is similar for each of the three cohorts.

## Discussion

Our study confirmed H1 replicating the effect that, compared to conditional probabilities, natural frequency formats boost insight and facilitate Bayesian reasoning without training [4–6]. Unlike what we expected in H2, after a 1-hour training intervention on how to translate probabilities into natural frequencies and how to use them to implement Bayes’ rule, posttest performance with conditional probabilities showed a more differentiated pattern compared to the pre-test conditions [7, 8]. Specifically, the agency-boosting training improved accurate test interpretation with conditional probabilities compared to no boost (H2a). However, the combination of training and conditional probabilities (the boost of agency) did not outperform the pretest condition where natural frequencies were given without training (the boost of insight) (H2b).

One explanation for this pattern could be that parts of our training intervention did not work. However, performance with conditional probabilities increased after the training, indicating that students understood how to translate conditional probabilities into natural frequencies. Also, performance with natural frequencies increased after the training, suggesting that students learned how to use natural frequencies to implement Bayes’ rule. This suggests that both aspects of the training intervention have worked, and that we should revisit Sedlmayer and Gigerenzer’s conclusion that after a training with natural frequencies “the degree of improvement…should be…larger than…without training” [7]. Specifically, our results suggest that a training on (a) how to translate conditional probabilities into natural frequencies and (b) how to implement them into Bayes’ rule is superior to no training [7, 8], but the training alone is *not* more effective than natural frequencies without training. In fact, the effect of natural frequencies used to boost insight before the training and the effect of training (without also facilitating insight) were equal in size. Future studies should investigate possible explanations for this pattern (e.g., difficulties translating conditional probabilities into natural frequencies) and whether the training may be further improved to increase its effectiveness.

Finally, maximal performance was achieved after the training *and* when information was formatted in natural frequencies. This supports H3. On average 89% of the students performed Bayesian calculations correctly under the condition combining both boosts. This result shows that the effect of natural frequencies as a boost of insight can and should be augmented with a training on how to use them (i.e., a boost of agency) and vice versa. More specifically, the finding of two main effects (training and format) without interaction suggests that the effects of natural frequencies as a boost for insight and a boost for agency are additive and that one intervention cannot be used to compensate for the other. This adds to similar findings in a parallel line of research on behavioral interventions called *nudges*, which, rather than conveying new insights or developing agency, exploit existing cognitive or behavioral tendencies (e.g., by setting a default to exploit people’s tendencies to stick with it) 31. Also for this type of intervention it was found that combining it with a boost of agency, that is, explicit explanations of why and how a behavior should be implemented, proves more effective than the nudge intervention alone [25, 26]. This suggests that the distinctions between boosts of insight, nudges, and boosts of agency are fruitful and more research on how to best combine these types of interventions in medical education is warranted.

With respect to the present study, we wanted to understand when to use which of these interventions to maximize the effective use of teaching resources and improve medical students’ test interpretation skills. Based on our results, we can state that a combined use of natural frequencies as boosts of insight (i.e., by making them the default for communicating test statistics) *and* as boosts of agency (i.e., by adopting short trainings on why and how to use natural frequencies) is the most effective approach to maximize accurate test interpretation.

### Strengths and Limitations

The current study was performed in an actual classroom setting in medical school. Although this increases external validity, the classroom setting did not allow us to systematically account for individual differences (e.g., related to prior exposure to statistics) or even individual characteristics such as age and gender (67 students chose not to provide information on either demographic variable). For lack of control over the educational setting, we could also not assess the presented relationships over longer follow-up periods and use control groups as in previous training studies [7, 8]. Although this study provides relevant first evidence as to the additive effect of natural frequencies––as a boost for insight and as an agency-boosting training––on medical students’ Bayesian reasoning abilities, future studies in more controlled settings should account for these limitations.

Given our pre-post study design, we cannot exclude that factors other than the training intervention may have influenced the results between pre- and posttest. But given the tight teaching schedule (see Table 1) and that pre- and posttest were administered directly before and after the training, history and maturation effects [24] seem unlikely. Similarly, we cannot exclude testing effects, that is, that simply using the same test questions during pre- and posttest may have had an effect on accurate test interpretation during the posttest. However, given that many physicians do not know how to accurately interpret test results using Bayes’ rule [16, 17], it seems implausible that a one-time exposure to two test questions can explain our findings and improve accurate test interpretation in 20 to 30% of the medical students as in our sample. Also, the fact that we found worse performance in only 1 to 2% of the participants suggests that regression to the mean effects, which are typical for pre-posttest designs [24], cannot explain the large performance improvements we found.

Finally, the test questions were taken from the Berlin Numeracy Test, a survey instrument cross-validated in various cultures and populations to measure numeracy, that is, people’s ability to reason with numbers [22]. Although both questions ask for a dichotomous outcome (i.e., membership: yes/no; poisonousness: yes/no), they use a different number of characteristics to describe the test population in the question using natural frequencies (i.e., binary gender: male/female) and the question with conditional probabilities (i.e., three colors: red/brown/white) (see Table 2). As a result, the question with natural frequencies may have been slightly easier to answer, artificially inflating the observed advantage of this format compared to conditional probabilities (with and without training). On the other hand, a meta-analysis, which reviewed all available studies comparing Bayesian reasoning tasks with two versus three population characteristics, estimated that this difference has “a negligible effect on responses in the conditional probability format” [27]. Thus, given the large effect sizes of the observed differences between the interventions, this limitation should not invalidate the main findings.

### Practical implications

Previous research using the Berlin Numeracy Test has shown that statistical literacy tends to be higher among medical students and professionals compared to those in other fields [28]. However, when it comes to medical test interpretation, performance of health professionals seems to be low on average and unrelated to the level of experience [29]. Thus, to improve accurate test interpretation, natural frequencies can and should be used to boost insight *and* agency of both medical students and more experienced physicians. Until more evidence becomes available, we hope that our findings (a) encourage policy makers to mandate the use of natural frequencies formats to communicate test statistics in medical school and in test manuals to boost (future) health professionals’ insight into test statistics and help interpret them more accurately. And (b), we hope our results convince educators to implement similar or adapted training interventions that teach (future) health professionals how to translate probabilities into natural frequencies and boost their skills for accurate test interpretation in medical schools and/or continuing medical education. From a policy perspective, it will likely take longer to widely implement natural frequencies as the standard for communicating test statistics. Thus, trainings seem to be the first important step toward empowering health professionals to accurately interpret test results in the short-term. All teaching and assessment materials are open access and can be obtained from the authors. Although the training intervention was designed to train undergraduate medical students, the materials can be easily adapted to different levels of expertise and medical specialties by changing the tests used in step 3 of the training (see Table 1).

## Conclusions

Neither boosting insight nor agency alone seem to suffice to maximally increase accurate test interpretation with natural frequencies. Natural frequencies should rather be used to boost insight *and* agency to maximize effective use of teaching resources. Thus, mandating the use of natural frequencies in test manuals *and* using them as didactical formats in training interventions provides the most effective way to enable more healthcare professionals to accurately interpret test results and, ultimately, to discuss them with their patients and meet the basic requirements of EBM.

## Data Availability

The datasets used and/or analysed during the current study are available from the corresponding author on reasonable request.

## Footnotes

1 We shall mention that Hertwig and Grüne-Yanhoff [30] have referred to the representation of test statistics in natural frequencies as a *short-term boost* that increases competencies in a specific context but not beyond a given set of problems. They further distinguished short-term boosts from *long-term boosts*, such as teaching people how to represent test statistics as natural frequencies. To avoid confusion, we acknowledge this parallel conceptual distinction here but prefer to use the terms ‘boosting of insight’ and ‘boosting of agency’ instead, because they are more explanatory, and we did not intend to test short term and long term effects in this study.

2 The two additional items assess other statistical competencies and were thus not used for this study.

3 We excluded 42 students from the analysis who came late for class or left early and thus could not participate in both tests and missed parts of the training. Of these excluded students, 6 indicated no sex or age. The remaining students we excluded were slightly more likely to be female (67% were female) than those included in the analysis (61% were female) but similar in terms of age (mean = 25.8 vs. 25.2; SD = 3.3 vs. 3.1; range = 22–33 vs. 21–42).
